# Indeterminate CT reporting of adult acute appendicitis: radiologist versus lexicon-based diagnostic certainty

**DOI:** 10.1186/s13244-026-02352-y

**Published:** 2026-07-22

**Authors:** Piyachai Siriphiphatcharoen, Rathachai Kaewlai, Sasima Tongsai, Jitti Chatpuwaphat, Shanigarn Thiravit, Papasorn Wattanakul, Patcharaporn Thaisuriyo, Napakadol Noppakunsomboon

**Affiliations:** 1 Department of Biochemistry, Faculty of Medicine Siriraj Hospital, Bangkok, Thailand; 2 Department of Radiology, Faculty of Medicine Siriraj Hospital, Bangkok, Thailand; 3 Department of Research, Faculty of Medicine Siriraj Hospital, Bangkok, Thailand; 4 Department of Undergraduate Education, Faculty of Medicine Siriraj Hospital, Bangkok, Thailand; 5 Department of Surgery, Faculty of Medicine Siriraj Hospital, Bangkok, Thailand

**Keywords:** Adult, Appendicitis, Multidetector computed tomography, Radiologists, Reference standards

## Abstract

**Objectives:**

To evaluate report indeterminacy, diagnostic performance, and agreement with final diagnosis in CT performed for suspected adult acute appendicitis (AA), comparing original radiologist reports, adjudicated impressions, and certainty derived from structured lexicon-based schemes. We also aimed to identify factors associated with indeterminate reporting.

**Materials and methods:**

A total of 983 consecutive CT reports between August 2021 and July 2025 were reviewed. Diagnostic certainty was categorized from original radiology reports (negative, indeterminate, positive), with indeterminate cases adjudicated by two radiologists. Two objective lexicon-based schemes were applied using extracted CT findings (appendiceal diameter (6-mm and 7-mm cutoffs), wall thickening, wall hyperenhancement, and periappendiceal fat stranding) to generate four certainty categories (negative, possible, probable, positive). Surgical, pathological, and clinical outcomes served as reference standards.

**Results:**

Acute appendicitis was confirmed in 393 patients (40%). Indeterminate reporting occurred in 15.5% of original reports, 12% of adjudicated reports, and 27.9–46.1% of lexicon-based schemes. Across all approaches, AA prevalence increased consistently with higher diagnostic certainty. Adjudicated reports demonstrated the highest diagnostic performance (sensitivity 85.2–98.7%, specificity 85.9–97.1%, accuracy 91.0–92.4%) and excellent agreement with final diagnosis (kappa = 0.819–0.838). Several CT features were independently associated with indeterminate reporting.

**Conclusions:**

Radiologist-adjudicated impressions conveyed diagnostic certainty more effectively than original reports and lexicon-based schemes, with lower indeterminacy and stronger agreement with the final diagnosis. These findings underscore the practical importance of careful wording and judicious use of indeterminate categories when communicating CT findings for suspected adult AA.

**Critical relevance statement:**

Careful wording of CT impressions, grounded in radiologists’ integrated judgment, can reduce diagnostic ambiguity and potentially improve clinical decision-making, supporting standardized languages to express diagnostic certainty in suspected adult acute appendicitis.

**Key Points:**

- CT reports for suspected appendicitis vary in wording, leading to indeterminate impressions, potentially affecting diagnostic performance and agreement with final diagnosis.
- Adjudicated radiologist impressions reduced indeterminate reports and achieved higher accuracy and agreement with final diagnosis than original reports or lexicon-based schemes.

**Graphical Abstract:**

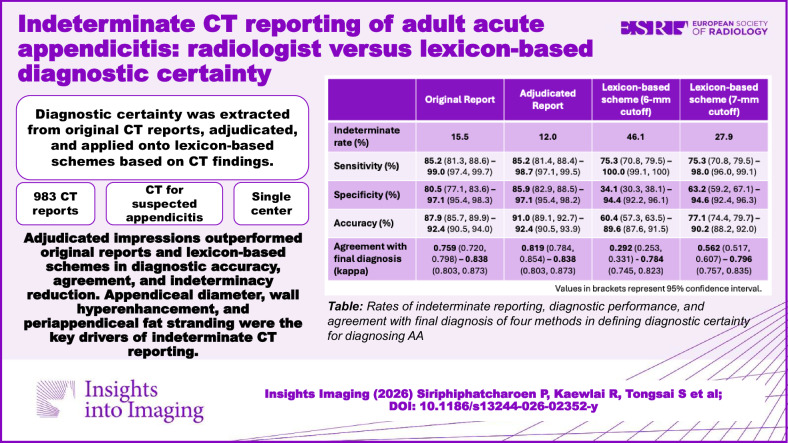

## Introduction

Computed tomography (CT) is the primary imaging modality for evaluating suspected acute appendicitis (AA) in non-pregnant adults in many institutions [1] and provides high diagnostic accuracy in most cases [2, 3]. Although ultrasound remains the first-line examination in centers with dedicated expertise in this technique, CT has broad accessibility and reproducibility. The majority of CT examinations allow confident confirmation or exclusion of AA; however, a non-trivial subset of studies is reported as indeterminate or equivocal. Prior work suggests that approximately 2–15% of CT reports fall into this category, and importantly, a substantial proportion of these indeterminate cases are ultimately diagnosed as AA [4–6]. When CT findings are inconclusive, clinical decision-making becomes more complex, and management pathways may vary considerably across providers [3, 5].

Clear communication of diagnostic certainty in radiology reports is increasingly recognized as a critical component of effective interdisciplinary care. Wording used to express diagnostic confidence and a clear understanding of the meaning of these phrases can influence clinical decision-making [7]. The American College of Radiology (ACR) has advocated for standardized terminology to convey diagnostic certainty, aiming to reduce ambiguity and improve report actionability [8]. However, while such expressions reflect radiologists’ integrated judgment, reliance on nuanced language alone may introduce variability across readers, experience levels, and practice settings.

One strategy to strengthen and standardize certainty expression is to anchor diagnostic confidence to predefined imaging features. In other disease domains, lexicon-based frameworks such as the Reporting and Data Systems (-RADS) link specific imaging findings to graded diagnostic certainty, improving consistency and communication [9]. In acute appendicitis, Leite et al proposed a structured CT lexicon combining appendiceal diameter and inflammatory findings to stratify disease likelihood [10]. The real-world performance of such feature-based approaches, particularly relative to radiologists’ synthesized impressions, remains insufficiently characterized. It is therefore unclear how these different methods influence indeterminate reporting rates, diagnostic performance, agreement with final diagnosis, and the imaging features that drive uncertainty.

Accordingly, this study compared diagnostic certainty across original radiologist impressions, adjudicated impressions, and structured lexicon-based schemes in CT performed for suspected adult AA. We assessed indeterminate reporting rates, diagnostic performance, agreement with final diagnosis, and factors associated with indeterminate interpretations.

## Materials and methods

### Study design and patient selection

**Fig. 1:**
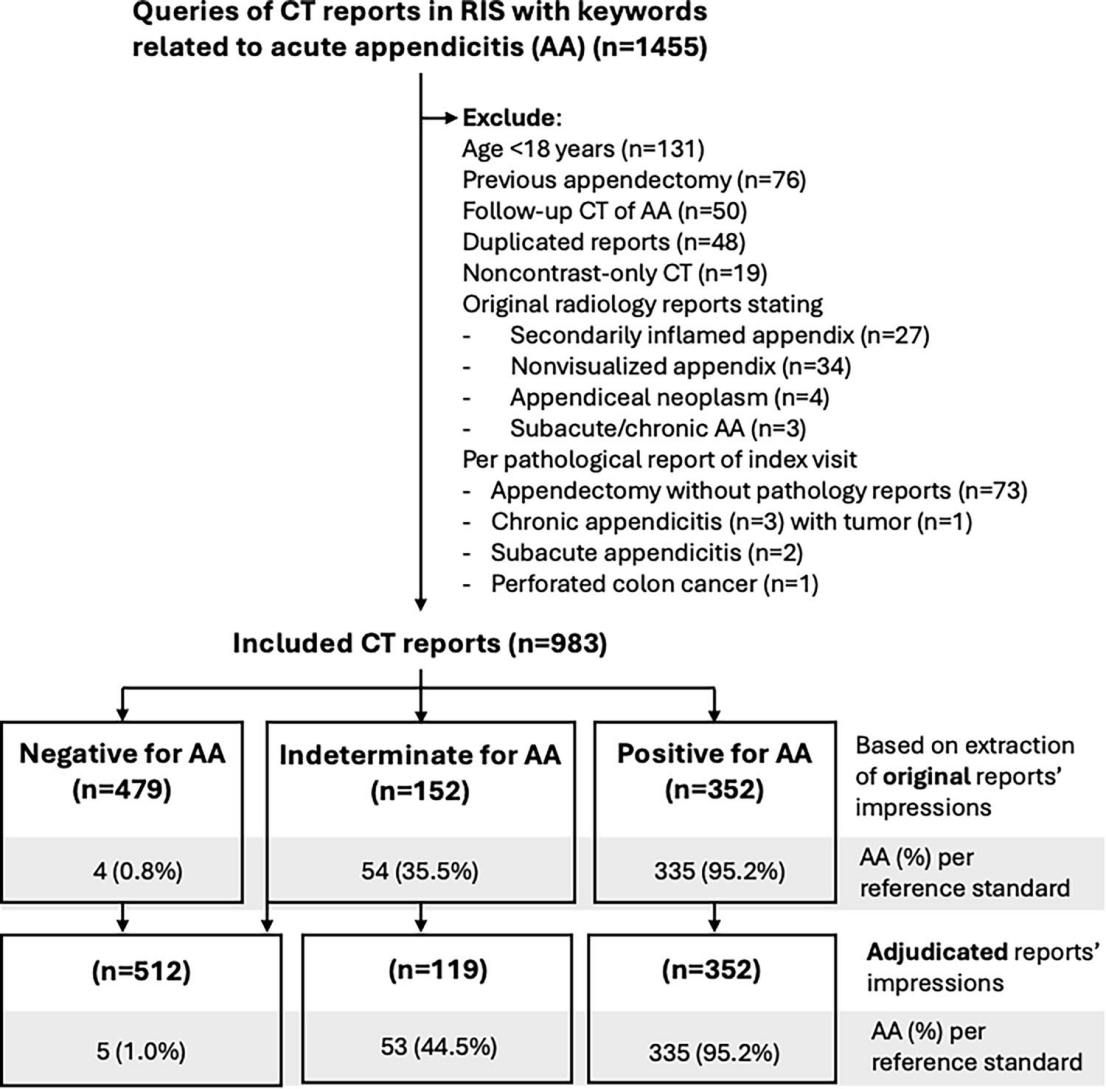
Flowchart of patient inclusion

This retrospective investigation was conducted at a 2200-bed urban academic medical center. Institutional Review Board approval was obtained (Protocol Number 696/2568(IRB4); Certificate of Approval Number Si 677/2025). As part of routine clinical care, all patients provided informed consent prior to undergoing CT. The requirement for additional research-specific informed consent was waived, given the retrospective design and minimal risk nature of the study. A search of the institutional Radiology Information System was performed by a departmental IT specialist under investigator supervision, using the key terms “appendicitis” and “right lower quadrant/RLQ pain/tenderness,” yielding 1455 reports of abdominopelvic CT examinations performed between August 2021 and July 2025. CT reports were eligible for inclusion if they were issued for adult patients (≥ 18 years) presenting with suspected AA and contained an original radiologist impression. Reports were excluded if patients had a history of prior appendectomy, if the CT was performed as a follow-up for known AA, or if the report was duplicated. Additional exclusion criteria based on CT characteristics included noncontrast-only examinations. Reports were further excluded if the radiologist’s impression indicated a secondarily inflamed appendix, nonvisualized appendix, appendiceal neoplasm, or subacute/chronic AA. Finally, cases were excluded based on pathological findings at the index visit, including appendectomy without available pathology reports, chronic appendicitis with or without tumor, subacute appendicitis, and perforated colon cancer. The full selection process is illustrated in Fig. 1. It should be noted that our institution does not follow a standardized imaging pathway for suspected AA, with ultrasound performed based on referring physician preference rather than as a mandatory first-line examination.

### Reporting radiologists

All included official radiology reports were issued by 39 board-certified radiologists as part of routine clinical care. Radiologists specialized in abdominal radiology (*n* = 11; 682 reports), body imaging (*n* = 18; 120 reports), or emergency radiology (*n* = 3; 169 reports); the remaining 12 reports were issued by radiologists undertaking body imaging fellowship training who are board-certified in radiology. Subspecialty classification was determined by a combination of formal fellowship training and departmental assignment. Years of experience ranged from 2 to 42 years (median 10 years). All reports were generated independently as part of routine clinical practice, and the reporting radiologists were not aware of the study hypothesis at the time of reporting. Radiologists were categorized by subspecialty to examine whether subspecialty background was associated with indeterminate reporting rates.

### Extraction of CT report determinacy and imaging findings

One study author (R.K.; emergency radiologist with 25-year experience) extracted the report data while blinded to the final diagnosis and outcomes.

1. **Determinacy of AA**: Each CT report was classified into three categories—negative, indeterminate, or positive for AA—based solely on the impression section. Classification was performed by one study author (R.K.), who systematically identified the presence of specific terminology related to AA as listed below. When the impression contained unambiguous language clearly fitting one category, that category was assigned directly. When the language was unclear, mixed, or did not map cleanly to a single category, the report was conservatively flagged as indeterminate, as the classifier was instructed to err toward indeterminate rather than force an assignment of negative or positive in uncertain cases. The classification was performed in a single pass by this author; indeterminate cases were subsequently reviewed in a separate adjudication process described below.

- *Negative*: normal appendix, unremarkable appendix, no evidence of appendicitis.
- *Indeterminate*: borderline, cannot be excluded, concerning, could be, enlarged appendix (without specifying appendicitis), equivocal, less likely, not typical, possible, probable, or suspected.
- *Positive*: likely, positive for, suggestive for, consistent with appendicitis, or those stating “appendicitis” without qualifiers.

Phrases such as “correlate with clinical context” or similar wording were also recorded. For this study, these phrases were analyzed as independent variables and were not used for determinacy categorization.

2. **CT findings**: Extracted imaging features (Supplementary Table S1) included appendiceal diameter, luminal gas, wall thickening, wall hyperenhancement, periappendiceal fat stranding, and other relevant findings (periappendiceal fluid collection, extraluminal air, hypoenhancing appendiceal wall, appendicolith, and masslike lesion/phlegmon). All findings except appendiceal diameter were recorded as present or absent; features not mentioned in the report were considered absent. Appendiceal diameter was measured directly from CT images by R.K. when not explicitly reported (*n* = 167) or described only as “normal” (*n* = 108). Alternative diagnoses proposed by the verifying radiologist were also recorded.

### Adjudication of indeterminate CT reports

**Fig. 2:**
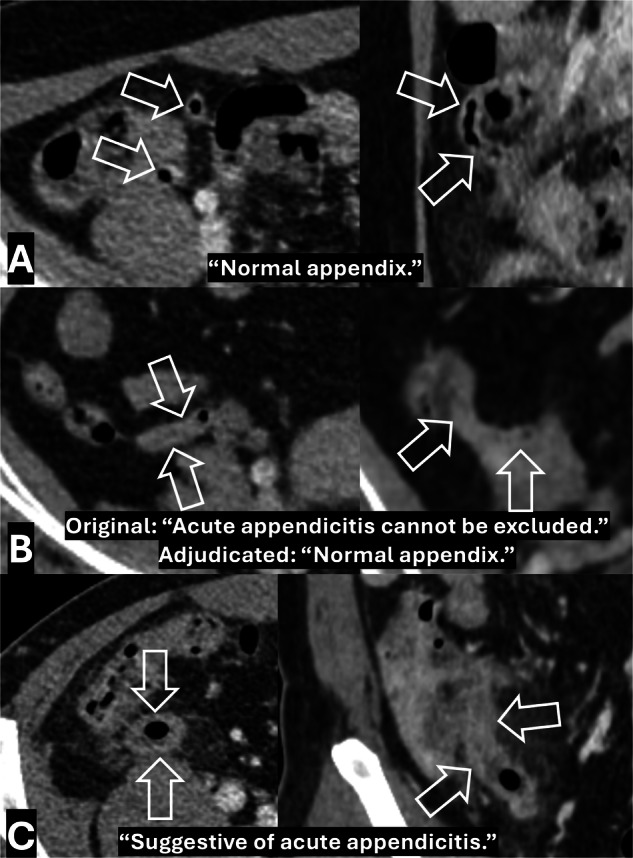
Examples of cases with (**A**) a normal appendix and unambiguous report language “normal appendix,” (**B**) borderline-sized appendix (7 mm; lack of air filling) and ambiguous report language “acute appendicitis cannot be excluded” that had been adjudicated as “normal appendix,” and (**C**) acute appendicitis and unambiguous report language “suggestive of acute appendicitis.” It should be emphasized that the adjudicators reviewed only the CT report for the purpose of adjudication but not these images. Arrows = appendices

CT reports originally classified as indeterminate were independently rereviewed by two radiologists (J.C., an emergency radiologist with 10 years of experience, and S.T., an abdominal radiologist with 13 years of experience). Importantly, adjudication involved re-assessment of the written radiology report only—specifically the impression section and full report text—and did not involve re-review or re-interpretation of the CT images themselves. Adjudication was limited to originally indeterminate reports, as these represented cases where diagnostic certainty could not be inferred from the impression alone; negative and positive reports were considered to contain sufficiently unambiguous language to preclude the need for re-review. Reviewers assessed the impression section and referred to the full report to determine whether the original verifying radiologist’s intended diagnostic certainty could be inferred. Each report was reclassified into three diagnostic certainty categories: negative (< 10% likelihood of AA), indeterminate (10–90%), and positive (> 90%). Consensus classification was assigned when both reviewers agreed on negative and positive findings. If they disagreed, the reports were classified as indeterminate. We acknowledge that a subset of originally negative or positive reports may have contained nuanced wording that adjudication could have reclassified. Examples of original and adjudicated reports, and a figure are provided in Supplementary Table S2 and Fig. 2, respectively.

### Categorization using lexicon-based schemes

Extracted CT findings were mapped into a lexicon-based diagnostic scheme proposed by Leite et al in 2005 [10]. The lexicon incorporates appendiceal diameter (< 6 mm, 6–10 mm, and > 10 mm) and the presence of three additional findings (wall thickening, wall hyperenhancement, and periappendiceal fat stranding) to classify studies into four certainty categories for AA: excludes, possible, probable, and definite. In the original scheme, a “completely gas-filled appendix” was also considered an excluding feature; however, this criterion was not applied in the present study. To allow more inclusive combinations of imaging findings, four subgroups were added to the possible category and two subgroups to the probable category. Given the increasing adoption of a 7-mm appendiceal diameter threshold for CT diagnosis of AA in contemporary practice [11], the lexicon was further modified by substituting a 7-mm cutoff for the original 6-mm threshold. The final lexicon-based schemes used in this investigation area are detailed in Supplementary Table S3.

### Clinical, surgical, and pathological data and reference standards

For patients managed nonoperatively, AA was defined based on multidisciplinary clinical consensus documented at discharge, reflecting contemporary management of AA. These patients typically had complicated AA managed with antibiotics and/or percutaneous drainage or were deemed unsuitable for surgery and treated conservatively. At our institution, appendectomy remains the standard treatment for AA. Final alternative diagnoses in patients without AA were determined based on discharge summaries.

### Outcome measures

The study evaluated original reports, adjudicated reports, and lexicon-based reports (6-mm and 7-mm versions) for (1) their ability to scale the likelihood of AA, assessed by AA rates across diagnostic certainty categories (3–4 categories: negative, indeterminate (possible or probable), and positive); and (2) overall diagnostic performance using reference standards.

Because the evaluated CT reports contained multiple certainty categories, diagnostic performance was calculated using predefined category groupings as follows:

- Original and adjudicated reports: With three certainty categories, performance was assessed using two dichotomizations: negative vs. non-negative (indeterminate + positive), and positive vs. non-positive (negative + indeterminate).
- Lexicon-based reports: With four certainty categories, performance was assessed using three dichotomizations: negative vs. non-negative (possible + probable + positive), positive vs. non-positive (negative + possible + probable), and low likelihood (negative + possible) vs. high likelihood (probable + positive).

Additionally, factors associated with report indeterminacy were evaluated for adjudicated reports.

### Statistical analysis

Descriptive statistics were used to summarize patient characteristics and CT report classifications. Continuous variables are presented as median with range, and categorical variables as frequencies and percentages. Differences in report distributions and rates of correct diagnosis among CT report categorization approaches, including original reports, adjudicated reports, and lexicon-based reports using 6-mm and 7-mm thresholds, were evaluated using appropriate comparative statistical tests.

Diagnostic test performance metrics, including sensitivity, specificity, positive and negative likelihood ratios, positive and negative predictive values, and overall accuracy, were calculated for each categorization method using the final clinical diagnosis as the reference standard. Agreement between CT report categorization methods and the final diagnosis was assessed using Cohen’s kappa statistic.

Clinical and imaging characteristics were compared across adjudicated CT report categories (negative, indeterminate, and definite) using the Pearson chi-square test for categorical variables and the Kruskal–Wallis test for continuous variables. When overall differences were significant, pairwise comparisons were performed with Bonferroni adjustment for multiple testing.

Multivariable associations between clinical and imaging variables and diagnostic category were assessed using multinomial logistic regression, with diagnostic category modeled as a nominal outcome and indeterminate specified as the reference category. Candidate variables for the multivariable multinomial logistic regression model were selected from univariable analyses based on a *p*-value < 0.10 for at least one outcome category, and age and sex were retained a priori. Categorical variables were entered using dummy coding.

Department was treated as a three-level institutional variable and evaluated using an overall Wald chi-square test, with category-specific estimates reported in the Supplementary Material. Variables with sparse data or evidence of quasi-complete separation were excluded. Adjusted odds ratios with 95% confidence intervals were reported. Sensitivity analyses excluding the department were conducted to assess model robustness.

Inter-rater agreement between radiologists was assessed using linear weighted Cohen’s kappa with 95% confidence intervals. All tests were two-sided, with *p* < 0.05 considered statistically significant. Analyses were performed using SPSS Statistics (IBM Corp).

## Results

After exclusion, a total of 983 radiology reports remained in the final cohort (Fig. 1). Among 983 patients (with an equal number of CT reports), most were female (64.9%), with a mean age of 45 years (range, 18–98 years). A final diagnosis of AA was established in 393 patients (40%). Appendectomy was performed in 433 patients (44%), yielding a negative appendectomy rate of 9.2%. Most CT examinations were performed during office hours (702/983; 71.4%) and interpreted by abdominal radiologists (682/983; 69.4%).

### Adjudicating the original CT reports

Original reports were classified as negative (*n* = 479; 48.7%), indeterminate (*n* = 152; 15.5%), or positive (*n* = 352; 35.8%). Adjudication by two radiologists demonstrated substantial inter-rater agreement (Cohen’s kappa of 0.711 (95% CI, 0.626, 0.796)). Adjudication reduced the number of indeterminate reports from 152 (15.5%) to 119 (12%) by increasing the negative reports from 479 to 512, while retaining the number of positive reports. Changes in report categorization after adjudication are illustrated in Fig. 1.

### Rate of indeterminate reports and scaling of AA likelihood

Indeterminate report rates were 15.5% for the original reports and 12% for the adjudicated reports. Lexicon-based schemes yielded substantially higher indeterminacy when combining the possible and probable categories: 46.1% (*n* = 453) using the 6-mm version and 27.9% (*n* = 274) using the 7-mm version. Across all four approaches, AA prevalence increased consistently with ascending certainty category. In original reports, AA was confirmed in 0.8% of negative, 35.5% of indeterminate, and 95.2% of positive cases. Adjudicated reports showed a similar pattern (1.0%, 44.5%, and 95.2%, respectively), with a slight reduction in indeterminate cases compared to original reports. For the 6-mm lexicon, AA prevalence rose from 0% (negative) to 12.5% (possible), 70.0% (probable), and 90.0% (positive). The 7-mm lexicon showed a comparable gradient: 2.1%, 19.4%, 76.2%, and 90.2%, respectively. Full distributions and AA proportions across all approaches are presented in Table 1.

**Table 1:** Comparison of original reports, adjudicated reports, and lexicon-based reports (6-mm and 7-mm versions) in categorizing CT reports for likelihood of acute appendicitis and their rates of correct diagnosis (*n* = 983)

	Number of patients (% within same grouping)	Number of patients with acute appendicitis (% within same grouping)
Original reports		
Negative	479 (48.7)	4 (0.8)
Indeterminate	152 (15.5)	54 (35.5)
Positive	352 (35.8)	335 (95.2)
Adjudicated reports^a^		
Negative	512 (52.1)	5 (1.0)
Indeterminate	119 (12.0)	53 (44.5)
Positive	352 (35.8)	335 (95.2)
Lexicon-based reports (6-mm version)		
Negative	201 (20.4)	0
Possible	383 (39.0)	48 (12.5)
Probable	70 (7.1)	49 (70.0)
Positive	329 (33.5)	296 (90.0)
Lexicon-based reports (7-mm version)		
Negative	381 (38.8)	8 (2.1)
Possible	211 (21.5)	41 (19.4)
Probable	63 (6.4)	48 (76.2)
Positive	328 (33.4)	296 (90.2)

^a^ Indeterminate original reports were adjudicated by two radiologists with a substantial (linear weighted Cohen’s kappa of 0.711 (95% CI, 0.626, 0.796))

### Diagnostic performance and agreement with final diagnosis (Table 2, Supplementary Table S4, and Table 3)

Adjudicated reports demonstrated the highest diagnostic performance across all grouping schemes. For the negative vs. non-negative grouping, adjudicated reports achieved sensitivity of 98.7% (95% CI: 97.1–99.5), specificity of 85.9% (95% CI: 82.9–88.5), and accuracy of 91.0% (95% CI: 89.1–92.7). For the positive vs. non-positive grouping, sensitivity was 85.2% (95% CI: 81.4–88.4), specificity 97.1% (95% CI: 95.4–98.2), and accuracy 92.4% (95% CI: 90.5–93.9). Original reports performed comparably in the positive vs. non-positive grouping (sensitivity 85.2%, specificity 97.1%, accuracy 92.4%) but showed lower specificity in the negative vs. non-negative grouping (80.5%; 95% CI: 77.1–83.6) and overall accuracy of 87.9% (95% CI: 85.7–89.9). Differences in sensitivity, specificity, and accuracy between original and adjudicated reports were modest, with overlapping 95% confidence intervals across most grouping schemes, particularly for the positive vs. non-positive grouping, where performance was identical.

Among lexicon-based schemes, the 7-mm version consistently outperformed the 6-mm version. For the low vs. high likelihood grouping, the 7-mm version achieved sensitivity of 87.5% (95% CI: 83.9–90.6), specificity of 92.0% (95% CI: 89.6–94.1), and accuracy of 90.2% (95% CI: 88.2–92.0), compared to 87.8%, 90.9%, and 89.6%, respectively, for the 6-mm version. The 6-mm version showed perfect sensitivity (100%) in the negative vs. non-negative grouping but at the cost of very low specificity (34.1%), limiting its practical utility (Table 2 and Supplementary Table S4). Agreement analyses mirrored these trends (Table 3): adjudicated reports yielded the highest kappa values (0.819–0.838), followed by the original report (0.759–0.838), the 7-mm lexicon (0.562–0.796), and the 6-mm lexicon (0.292–0.784), with the low vs. high likelihood grouping performing best among lexicon-based schemes for both versions.

**Table 2:** Diagnostic test parameters of four methods of categorizing computed tomography reports for the likelihood of acute appendicitis based on different grouping schemes^a^ (*n* = 983)

	Sensitivity	Specificity	PPV	NPV	Accuracy
Original report					
Negative vs. non-negative	99.0 (97.4, 99.7)	80.5 (77.1, 83.6)	77.2 (74.2, 80.0)	99.2 (97.8, 99.7)	87.9 (85.7, 89.9)
Positive vs. non-positive	85.2 (81.3, 88.6)	97.1 (95.4, 98.3)	95.2 (92.5, 97.0)	90.8 (88.6, 92.6)	92.4 (90.5, 94.0)
Adjudicated report					
Negative vs. non-negative	98.7 (97.1, 99.5)	85.9 (82.9, 88.5)	82.4 (78.7, 85.6)	99.0 (97.7, 99.6)	91.0 (89.1, 92.7)
Positive vs. non-positive	85.2 (81.4, 88.4)	97.1 (95.4, 98.2)	95.2 (92.4, 97.0)	90.8 (88.3, 92.8)	92.4 (90.5, 93.9)
Lexicon-based diagnostic scheme (6-mm version)					
Negative vs. non-negative	100.0 (99.1, 100.0)	34.1 (30.3, 38.1)	50.3 (48.8, 51.7)	100.0 (98.2, 100.0)	60.4 (57.3, 63.5)
Low vs. high likelihood	87.8 (84.1, 90.9)	90.9 (88.2, 93.1)	86.5 (83.2, 89.2)	91.8 (89.5, 93.6)	89.6 (87.6, 91.5)
Positive vs. non-positive	75.3 (70.8, 79.5)	94.4 (92.2, 96.1)	90.0 (86.5, 92.6)	85.2 (82.8, 87.2)	86.8 (84.5, 88.8)
Lexicon-based diagnostic scheme (7-mm version)					
Negative vs. non-negative	98.0 (96.0, 99.1)	63.2 (59.2, 67.1)	64.0 (61.5, 66.4)	97.9 (95.9, 98.9)	77.1 (74.4, 79.7)
Low vs. high likelihood	87.5 (83.9, 90.6)	92.0 (89.6, 94.1)	88.0 (84.7, 90.6)	91.7 (89.5, 93.5)	90.2 (88.2, 92.0)
Positive vs. non-positive	75.3 (70.8, 79.5)	94.6 (92.4, 96.3)	90.2 (86.8, 92.9)	85.2 (82.9, 87.3)	86.9 (84.6, 88.9)

^a^ Original reports (3 probabilities of appendicitis) were regrouped into two categories of either (A) negative vs. indeterminate plus positive (“negative vs. non-negative”) or (B) negative plus indeterminate vs. positive (“positive vs. non-positive”). The rest of the reports (4 probabilities of appendicitis) were regrouped into two categories of (A) negative vs. the rest (“negative vs. non-negative”), (B) negative plus possible (“low likelihood”) vs. probable plus positive (“high likelihood”), and (C) positive vs. the rest (“positive vs. non-positive”)

**Table 3:** Agreements (Cohen’s kappa) between computed tomography reports and the final diagnosis of acute appendicitis

Methods of grouping	Original report	Adjudicated report	Lexicon-based report (6-mm version)	Lexicon-based report (7-mm version)
Negative vs. non-negative	0.759 (0.720, 0.798)	0.819 (0.784, 0.854)	0.292 (0.253, 0.331)	0.562 (0.517, 0.607)
Low vs. high likelihood	N/A	N/A	0.784 (0.745, 0.823)	0.796 (0.757, 0.835)
Positive vs. non-positive	0.838 (0.803, 0.873)	0.838 (0.803, 0.873)	0.717 (0.672, 0.762)	0.719 (0.674, 0.764)

### Factors associated with indeterminate report

Several clinical and imaging variables differed significantly across adjudicated report categories (Table 4). Patients with indeterminate reports were younger (median age 37 years) compared to those with positive reports (52.5 years) and negative reports (42 years; *p* < 0.001). Male sex was less frequent in indeterminate reports (24.4%) than in positive reports (46.3%; *p* < 0.001). Indeterminate reports were more likely to contain a “clinical correlation” statement (32.8%) compared to negative (6.3%) and positive reports (0.9%; *p* < 0.001). Laboratory values also differed significantly across reporting groups. White blood cell (WBC) count, neutrophil percentage, and neutrophil-to-lymphocyte ratio increased stepwise from negative through indeterminate to positive reports (all *p* < 0.001), with the highest values observed in the positive group.

**Table 4:** Comparison of characteristics of adjudicated CT reports deemed *indeterminate* and those negative or definite for acute appendicitis (*n* = 983)

Factors	All reports (*n* = 983)	Negative (*n* = 512)	Indeterminate (*n* = 119)	Positive (*n* = 352)	*p*-values
Median age (years, range)	45 (18, 98)	42 (18, 98)	37 (18, 92)	**52.5 (18, 92)**	< 0.001
Male sex	345 (35.1)	153 (29.9)	29 (24.4)	**163 (46.3)**	< 0.001
Final diagnoses No explanation of S&S Uncomplicated AA - Phlegmonous appendicitis - Appendicitis with serositis Complicated AA - Gangrenous appendicitis - Appendicitis w/ microperforation - Perforated appendicitis - Appendiceal abscess Other diagnoses	121 (12.3) 157 (16.0) 105 (10.7) 21 (2.1) 18 (1.8) 90 (9.2) 2 (0.2) 469 (47.7)	100 (19.5) **3 (0.6)** *1 (0.2)* 0 (0) 0 (0) *1 (0.2)* 0 (0) *407 (79.5)*	18 (15.1) 33 (27.7) *13 (10.9)* *1 (0.8)* 2 (1.7) *4 (3.4)* 0 (0) *48 (40.3)*	**3 (0.9)** 121 (34.4) *91 (25.9)* *20 (5.7)* 16 (4.5) *85 (24.1)* 2 (0.6) *14 (4.0)*	< 0.001
Laboratory values					
Median white blood cell (cells/µL, range) (*n* = 971)	11,900 (10, 56,680)	**10,840 (10, 56,680)**	12,500 (110, 30,600)	13,340 (1010, 41,460)	< 0.001
Mean neutrophil percentages (SD) (*n* = 971)	76.3 (13.4)	74.2 (13.5)	75.7 (15.1)	**79.5 (12.1)**	< 0.001
Median lymphocyte percentages (range)	12.5 (0.4, 90.9)	15.1 (0.4, 70.1)	14.1 (1.0, 90.9)	**10.5 (0.8, 84.0)**	< 0.001
Median neutrophil-to-lymphocyte ratio (*n* = 971)	6.3 (0.01, 248.8)	5.1 (0.2, 248.8)	5.7 (0.1, 93.0)	**7.9 (0.01, 121.8)**	< 0.001
**Operation for presumed AA**	433 (44.0)	*16 (3.1)*	*73 (61.3)*	*344 (97.7)*	< 0.001
Alternative diagnosis					< 0.001
Clinical	469 (47.7)	*407 (79.5)*	*48 (40.3)*	*14 (4.0)*	
CT reports	380 (38.7)	*325 (63.5)*	*36 (30.3)*	*19 (5.4)*	< 0.001
Ordering departments Emergency	(*n* = 970) 510 (52.6)	(*n* = 504) *238 (47.2)*	(*n* = 116) 63 (54.3)	(*n* = 350) *209 (59.7)*	< 0.001
Outpatient	339 (34.9)	185 (36.7)	36 (31.0)	118 (33.7)	
Inpatient	121 (12.5)	81 (16.1)	17 (14.7)	**23 (6.6)**	
**Ultrasound prior to CT**	45 (4.6)	25 (4.9%)	7 (5.9)	13 (3.7)	0.548
**CT during duty hours**	702 (71.4)	368 (71.9)	83 (69.7)	251 (71.3)	0.897
**CT coverage of abdomen/pelvis**	497 (50.6)	255 (49.8)	57 (47.9)	185 (52.6)	0.602
**Appendiceal diameter** (mm; median, range)	7 (2, 33)	*6 (2, 16)*	*8 (4, 21)*	*12 (6, 33)*	< 0.001
**Wall thickening**	287 (29.2)	*2 (0.4)*	*56 (47.1)*	*229 (65.1)*	< 0.001
**Wall hyperenhancement**	308 (31.3)	*2 (0.4)*	*60 (50.4)*	*246 (69.9)*	< 0.001
**Periappendiceal fat stranding**	396 (40.3)	*1 (0.2)*	*74 (62.2)*	*321 (91.2)*	< 0.001
**Appendicolith**	125 (12.7)	**16 (3.1)**	19 (16.0)	90 (25.6)	< 0.001
**Periappendiceal fluid collection**	56 (5.7)	*1 (0.2)*	*3 (2.5)*	*52 (14.8)*	< 0.001
**Extraluminal air**	22 (2.2)	0 (0)	3 (2.5)	19 (5.4)	< 0.001^b^
**Hypoenhancing appendix wall**	92 (9.4)	0 (0)	*5 (4.2)*	*87 (24.7)*	< 0.001
**Appendiceal mass or phlegmon**	3 (0.3)	0 (0)	0 (0)	3 (0.9)	0.067
**Cecal apical thickening**	25 (2.5)	0 (0)	6 (5.0)	19 (5.4)	< 0.001^b^
**Verifying radiologists**					0.495
Abdominal	682 (69.4)	357 (69.7)	81 (68.1)	244 (69.3)	
Body	120 (12.2)	66 (12.9)	19 (16.0)	35 (9.9)	
Emergency	169 (17.2)	83 (16.2)	17 (14.3)	69 (19.6)	
Body imaging fellow	12 (1.2)	6 (1.2)	2 (1.7)	4 (1.1)	
**Reports containing “clinical correlation” statement**	*74 (7.5)*	*32 (6.3)*	*39 (32.8)*	*3 (0.9)*	< 0.001
**CT to last follow-up (days)**	319 (0, 1541)	311 (0, 1518)	278 (0, 1541)	334 (0, 1513)	0.325

Significant values are underlined

Values in bold indicate values that exhibit a significant difference from the other two categories in a pairwise comparison

Values in italics indicate same-row pairs that exhibit a significant difference in a pairwise comparison

^a^ All pairwise comparisons fail to reach significance (*p* > 0.05)

^b^ Pairwise comparisons were not performed for categories with column proportions equal to zero or one due to insufficient variability for statistical testing

The use of a preceding ultrasound prior to CT did not differ significantly among the three reporting categories. Regarding CT findings, appendiceal diameter was significantly smaller in indeterminate reports (median 8 mm) than in positive reports (median 12 mm) but larger than in negative reports (median 6 mm; *p* < 0.001). Wall thickening (47.1%), wall hyperenhancement (50.4%), and periappendiceal fat stranding (62.2%) were present in a substantial proportion of indeterminate reports—intermediate between negative (0.4%, 0.4%, and 0.2%, respectively) and positive reports (65.1%, 69.9%, and 91.2%, respectively; all *p* < 0.001). Radiologist subspecialty distribution was similar across all three report categories—abdominal radiology accounted for approximately 69% of reports in each group—and the difference was not statistically significant (*p* = 0.495), suggesting that subspecialty background did not meaningfully influence the likelihood of indeterminate reporting.

On multivariable multinomial logistic regression (Table 5 and Supplementary Table S5), four CT features were independently associated with indeterminate reporting. Absence of periappendiceal fat stranding was the strongest predictor of a negative rather than indeterminate report (aOR 308.91; 95% CI: 35.63–2678.40; *p* < 0.001), followed by absence of wall hyperenhancement (aOR 144.37; 95% CI: 23.33–893.48; *p* < 0.001) and absence of wall thickening (aOR 21.04; 95% CI: 3.29–134.69; *p* = 0.001). Larger appendiceal diameter was independently associated with a positive rather than indeterminate report (aOR 1.487; 95% CI: 1.308–1.689; *p* < 0.001), while smaller diameter predicted a negative report (aOR 0.621; 95% CI: 0.503–0.766; *p* < 0.001). Absence of an alternative CT diagnosis strongly predicted a positive report over indeterminate (aOR 7.31; 95% CI: 3.10–17.24; *p* < 0.001). Male sex was also independently associated with a positive report (aOR 1.93; 95% CI: 1.05–3.55; *p* = 0.036). Older age independently predicted a negative report over indeterminate (aOR 1.035 per year; 95% CI: 1.010–1.061; *p* = 0.006). Absence of appendicolith was associated with a negative report (aOR 5.03; 95% CI: 1.30–19.50; *p* = 0.019). In an expanded multivariable analysis including prior ultrasonography and laboratory values, the main associations remained unchanged; prior ultrasonography was not independently associated with CT report category and did not meet criteria for retention in the final model (Supplementary Table S5).

**Table 5:** Univariable and multivariable multinomial logistic regression analyses for factors associated with indeterminate CT reports in adults suspected of acute appendicitis (reference: indeterminate report) (*n* = 119)

Predictor	Outcome	Univariable OR (95% CI)	*p*-value	Multivariable aOR (95% CI)	*p*-value
Age (per year)	Negative	1.006 (0.996, 1.016)	0.262	**1.035 (1.010, 1.061)**	**0.006**
	Definite	1.018 (1.007, 1.028)	< 0.001	1.007 (0.993, 1.021)	0.318
Appendix diameter	Negative	0.462 (0.405, 0.527)	< 0.001	**0.621 (0.503, 0.766)**	**< 0.001**
	Definite	1.484 (1.350, 1.632)	< 0.001	**1.487 (1.308, 1.689)**	**< 0.001**
Male sex	Negative	1.323 (0.836, 2.094)	0.233	1.134 (0.414, 3.108)	0.807
	Definite	2.677 (1.676, 4.274)	< 0.001	**1.925 (1.045, 3.546)**	**0.036**
No alternative diagnosis	Negative	0.250 (0.162, 0.384)	<0.001	0.692 (0.292, 1.643)	0.404
	Definite	7.602 (4.149, 13.929)	< 0.001	**7.309 (3.099, 17.239)**	**< 0.001**
No wall thickening	Negative	226.667 (53.999, 951.458)	< 0.001	**21.044 (3.288, 134.685)**	**0.001**
	Definite	0.477 (0.313, 0.728)	< 0.001	1.083 (0.597, 1.961)	0.794
No wall hyperenhancement	Negative	259.322 (61.788, 1088.366)	< 0.001	**144.370 (23.328, 893.479)**	**< 0.001**
	Definite	0.438 (0.286, 0.671)	< 0.001	**0.477 (0.259, 0.877)**	**0.017**
No periappendiceal fat stranding	Negative	840.311 (114.116, 6187.786)	< 0.001	**308.910 (35.628, 2678.399)**	**< 0.001**
	Definite	0.159 (0.094, 0.268)	< 0.001	**0.345 (0.164, 0.726)**	**0.005**
No appendicolith	Negative	5.890 (2.928, 11.848)	< 0.001	**5.031 (1.298, 19.503)**	**0.019**
	Definite	0.553 (0.320, 0.955)	0.033	0.777 (0.361, 1.675)	0.520
Department group	Negative	ns	0.349–0.615	ns	0.540–0.987
	Definite	Significant	0.011–0.017	Significant	0.015–0.080

Multivariable multinomial logistic regression was adjusted for age, sex, appendix diameter, alternative diagnosis on CT, wall thickening, wall hyperenhancement, periappendiceal fat stranding, appendicolith, and periappendiceal fluid collection. Variables with sparse data or evidence of quasi-complete separation were excluded from the final model. The reference outcome category was indeterminate

Significant *p*-values are underlined

Values in bold indicate values that exhibit a significant difference in a multivariable analysis

*aOR* adjusted odds ratio, *CI* confidence interval, *ns* not significant

## Discussion

In this investigation, we evaluated how different CT reporting approaches conveyed diagnostic certainty in suspected adult AA. Across all approaches, increasing levels of reported diagnostic certainty were associated with progressively higher AA prevalence, supporting the validity of certainty-based reporting. Reports based on radiologists’ synthesized impressions, particularly after adjudication, demonstrated the lowest indeterminate rates, highest diagnosis accuracy, and strongest agreement with final clinical diagnosis of all approaches evaluated.

Original reports showed an indeterminate rate of 15.5%, which decreased to 12.0% following adjudication. This reduction was achieved without re-reviewing CT images—adjudication involved only re-assessment of report wording against predefined certainty thresholds, isolating the effect of language and certainty expression rather than image interpretation itself. The fact that reclassification was possible through wording re-assessment alone suggests that a meaningful proportion of indeterminacy in our cohort reflected imprecise language rather than unavoidable diagnostic ambiguity. The practical implication is therefore directed at radiologists in routine practice: more deliberate and consistent use of certainty terminology, guided by a simplified three-tier framework, has the potential to reduce indeterminate reporting, decrease unnecessary surgical referral, and improve clinical communication—without requiring any change in image interpretation or additional reading steps. Importantly, although adjudication did not alter the number of positive reports, the redistribution of indeterminate cases toward the negative category carries direct clinical relevance. In our cohort, 61.3% of patients with indeterminate reports underwent surgery for presumed AA compared to only 3.1% of those with negative reports, suggesting that imprecise certainty language—rather than true diagnostic uncertainty—may have contributed to unnecessary surgical referral in some cases. More deliberate use of certainty terminology, therefore, has the potential to reduce unwarranted operations and improve clinical decision-making without any change in image interpretation. While differences in binary diagnostic performance metrics between original and adjudicated reports were modest and should be interpreted in the context of overlapping confidence intervals, the more meaningful gains were reflected in the reduction of indeterminate reporting rates and stronger agreement with final diagnosis, as evidenced by higher kappa values in adjudicated reports (0.819–0.838 vs. 0.759–0.838; Table 3). The substantial inter-rater agreement observed during adjudication (linear weighted kappa 0.711) supports the feasibility and reproducibility of a simplified three-tier certainty framework, serving as a proof-of-concept for its prospective adoption as a departmental reporting standard rather than a second-reading process. This aligns with prior literature supporting indeterminate reporting as a distinct third diagnostic category [5, 12–14], in contrast to five-tier certainty scales, which have shown wide variability in how intermediate tiers are interpreted and grouped [15–21].

Prior studies consistently report indeterminate CT interpretations in approximately 2–15% of cases [4–6]. Importantly, up to one-third of indeterminate cases are ultimately confirmed as AA—a proportion exceeded in our cohort (35.5–44.5% when utilizing a lexicon-based scheme), underscoring the clinical relevance of how uncertainty is communicated. Reported indeterminacy rates have remained relatively stable over time [5, 14–17, 19, 22], suggesting that diagnostic uncertainty represents a persistent and meaningful category rather than a purely technical limitation.

Non-binary reporting is well established in ultrasound evaluation of AA, where non-visualization of the appendix is common [23–25]. Although indeterminate assessment is less frequent on CT, graded certainty remains clinically relevant. Non-binary certainty frameworks allow more transparent communication of diagnostic probability and may be flexibly applied depending on clinical context, such as prioritizing sensitivity in screening settings or specificity when guiding operative decisions.

Lexicon-based reporting systems such as BI-RADS and LI-RADS have been highly successful in other disease domains [26, 27], promoting standardized terminology, improved communication, and research reproducibility. However, our findings suggest that direct translation of such rule-based approaches to CT diagnosis of AA may be less effective as these schemes incorporate a limited number of imaging features and do not reflect the nuanced weighting, internal consistency, and contextual judgment routinely applied by radiologists. Our results suggest that structured lexicon-based schemes may be better suited as adjuncts rather than replacements for expert interpretive judgment in AA. Among the two lexicon versions tested, the 7-mm diameter cutoff consistently outperformed the 6-mm version, with higher specificity and stronger agreement with final diagnosis across all grouping schemes, supporting its preferential use when a lexicon-based approach is applied.

Few prior studies have specifically examined factors associated with indeterminate CT reporting for AA. We identified several CT findings independently associated with indeterminate interpretations on multivariable analysis, namely the absence of periappendiceal fat stranding, the absence of wall hyperenhancement, and the absence of wall thickening being predictive of a negative rather than indeterminate report. Larger appendiceal diameter independently predicted a positive report over indeterminate, while the absence of an alternative CT diagnosis strongly favored a positive report. These findings are consistent with previous work identifying borderline or absent classic CT features as contributors to diagnostic uncertainty [4, 14, 21, 22, 28]. Wall hyperenhancement and periappendiceal fat stranding, although often reported in binary terms, are inherently subjective when mild and may be influenced by inter-radiologist variability in reporting threshold. Improved methods to quantify these features may facilitate future development of more standardized lexicons.

This study has several limitations. First, its retrospective, single-institution design may limit generalizability. Second, CT findings were extracted from original reports rather than image re-review, and unmentioned findings were assumed absent. Third, non-laboratory clinical data were not collected. Fourth, reference standards differed between operated and non-operated patients, introducing potential verification bias, though this composite standard reflects contemporary AA management. Fifth, the definition of indeterminate reporting is inherently subjective, and factors associated with indeterminate reporting may overlap with those contributing to diagnostic errors. Finally, adjudication was applied exclusively to indeterminate reports and is not directly applicable to routine practice; it was used as an analytic tool assuming radiologists might converge on similar certainty categories if standardized terminology were adopted—an assumption requiring prospective validation. The specific reasoning behind each adjudicated reclassification was not captured, and future studies using structured adjudication forms documenting the basis for each decision would allow more mechanistic insight into how holistic radiologist judgment differs from lexicon-based approaches.

In conclusion, CT reports that expressed diagnostic certainty using radiologists’ integrated interpretive judgment, particularly when standardized through adjudication, demonstrated lower indeterminate rates, higher diagnostic performance, and stronger agreement with final diagnosis than lexicon-based approaches. These findings support efforts to standardize certainty language in CT reporting for suspected adult AA while preserving expert judgment to enhance clinical communication and decision-making.

## Supplementary information

ELECTRONIC SUPPLEMENTARY MATERIAL

## Data Availability

The data used to support the findings of this study are included in the article. The raw data of this manuscript are available upon reasonable request.

## Abbreviations

AA: Acute appendicitis
ACR: American College of Radiology
CT: Computed tomography
RADS: Reporting and Data Systems
