# Supplementary material for: Indeterminate CT reporting of adult acute appendicitis: radiologist versus lexicon-based diagnostic certainty
Source: Insights Imaging. 2026 Jul 22;17:189. doi: 10.1186/s13244-026-02352-y (PMC13391980; doi:10.1186/s13244-026-02352-y)
Supplement: Supplementary file 1 — ELECTRONIC SUPPLEMENTARY MATERIAL [file 13244_2026_2352_MOESM1_ESM.pdf]

Indeterminate CT reporting of adult acute appendicitis: radiologist versus lexicon-based diagnostic certainty

ELECTRONIC SUPPLEMENTARY MATERIAL

Supplementary Materials and Methods:

Study design and patient selection

This retrospective investigation was conducted at a 2,200-bed urban academic medical center. Institutional Review Board approval was obtained (Protocol Number 696/2568(IRB4); Certificate of Approval Number Si 677/2025). As part of routine clinical care, all patients provided informed consent prior to undergoing CT. The requirement for additional research-specific informed consent was waived given the retrospective design and minimal risk nature of the study. A dedicated search of the institutional radiology information system was performed by one of the authors (RK; emergency radiologist with 25-year experience) using the key terms "appendicitis" and "right lower quadrant/RLQ pain/tenderness", yielding 1,455 reports of abdominopelvic CT examinations performed between August 2021 and July 2025. CT reports were eligible for inclusion if they were issued for adult patients ($\geq$18 years) presenting with suspected AA and contained an original radiologist impression. Reports were excluded if patients had a history of prior appendectomy, if the CT was performed as a follow-up for known AA, or if the report was duplicated. Additional exclusion criteria based on CT characteristics included noncontrast-only examinations. Reports were further excluded if the radiologist's impression indicated a secondarily inflamed appendix, nonvisualized appendix, appendiceal neoplasm, or subacute/chronic AA. Finally, cases were excluded based on pathological findings at the index visit, including appendectomy without available pathology reports, chronic appendicitis with or without tumor, subacute appendicitis, and perforated colon cancer. The full selection process is illustrated in **Figure 1**.

CT acquisitions

All included CT examinations were performed with intravenous contrast administration on one of three CT scanners (two 256-slice Revolution Apex scanners and either a 64-slice Discovery CT750 HD or LightSpeed VCT scanners; all from GE Healthcare). Scan parameters were set at 300 mAs for the 64-slice scanners and 250 mAs for the 256-slice scanners, with a tube potential of 120 kVp and a rotation time of 0.5 seconds. The pitch and detector coverage were 1.375:1 and 40 mm for 64-slice scanners, and 0.992:1 and 80 mm for the 256-slice scanners. Scans were acquired at the thinnest available slice thickness (0.625 mm or 1.25 mm), and axial images of 1.25-mm slice thickness were reconstructed and transferred to the Picture Archiving and Communication Systems (PACS). Official radiology reports were available for all patients and were issued by 39 radiologists specializing in abdominal radiology (682 reports), body imaging (120 reports), emergency radiology (169 reports), or by body imaging fellows in training (12 reports).

Clinical, surgical, pathological data, and reference standards

The Acute Care Surgery Division database was cross-checked to ensure complete capture of patients diagnosed with AA during the study period. No additional cases were identified beyond those retrieved from the radiology report database. Patients who underwent CT examinations at outside hospitals prior to transfer were excluded.

Clinical data, including surgical and pathological reports and discharge summaries, were obtained from the electronic medical records. In patients who underwent appendectomy, the diagnosis of AA was confirmed by pathological examination. Subacute appendicitis, chronic appendicitis, periappendicitis, and lymphoid hyperplasia were classified as negative for AA.

For patients managed nonoperatively, AA was defined based on multidisciplinary clinical consensus documented at discharge, reflecting contemporary management of AA. These patients typically had complicated AA managed with antibiotics and/or percutaneous drainage or were deemed unsuitable for surgery and treated conservatively. At our institution, appendectomy remains the standard treatment for AA. Final alternative diagnoses in patients without AA were determined based on discharge summaries.

Supplementary Results

In an expanded multinomial logistic regression analysis including additional clinical variables, several factors remained independently associated with CT report classification (Supplementary Table S5). Compared with indeterminate reports, negative CT reports were independently associated with smaller appendix diameter (adjusted odds ratio [aOR] 0.61, 95% confidence interval [CI] 0.49–0.76; p<0.001), older age (aOR 1.03 per year, 95% CI 1.01–1.06; p=0.008), absence of wall thickening (aOR 18.19, 95% CI 2.88–115.07; p=0.002), absence of wall hyperenhancement (aOR 147.74, 95% CI 23.32–935.93; p<0.001), absence of periappendiceal fat stranding (aOR 277.51, 95% CI 32.00–2406.86; p<0.001), and absence of appendicolith (aOR 5.57, 95% CI 1.34–23.11; p=0.018). Definite CT reports were independently associated with larger appendix diameter (aOR 1.47, 95% CI 1.30–1.68; p<0.001), male sex (aOR 1.89, 95% CI 1.02–3.51; p=0.045), absence of an alternative diagnosis on CT (aOR 7.33, 95% CI 3.11–17.29; p<0.001), wall hyperenhancement (aOR 2.13, 95% CI 1.14–3.85; p=0.017), periappendiceal fat stranding (aOR 2.94, 95% CI 1.37–6.25; p=0.005), and outpatient department evaluation (aOR 3.75, 95% CI 1.32–10.66; p=0.013). Ultrasound prior to CT was not retained in the final multivariable model.

Supplementary Table S1: Operation definitions of extracted CT findings

CT findings	Definition	Recording
**Appendiceal diameter**	Outer wall-to-outer wall measurement of the appendix at its widest point on axial image, excluding any appendicolith	Continuous (mm); measured by RK when not explicitly reported (n=167) or described only as "normal" (n=108)
**Luminal gas**	Presence of gas within the appendiceal lumen	Binary (present/absent)
**Wall thickening**	Appendiceal wall thickness >2-3 m or as described as thickened by the reporting radiologist	Binary (present/absent)
**Wall hyperenhancement**	Increased enhancement of the appendiceal wall relative to adjacent bowel on contrast-enhanced CT, or described as hyper enhancing by the reporting radiologist	Binary (present/absent)
**Periappendiceal fat stranding**	Increased attenuation of periappendiceal mesenteric fat on CT, or described as fat stranding/dirty fat by the reporting radiologist	Binary (present/absent)
**Periappendiceal fluid collection**	Discrete fluid collection adjacent to the appendix, distinct from free fluid	Binary (present/absent)
**Extraluminal air**	Gas outside the appendiceal lumen in the periappendiceal region, suggesting perforation	Binary (present/absent)
**Hypoenhancing appendiceal wall**	Focal or diffuse decreased or absent wall enhancement on contrast-enhanced CT, suggesting ischemia or necrosis	Binary (present/absent)
**Appendicolith**	Calcified or hyperdense intraluminal appendiceal deposit	Binary (present/absent)
**Masslike lesion/phlegmon**	Ill-defined soft tissue density mass or inflammatory conglomerate in the right lower quadrant involving or obscuring the appendix	Binary (present/absent)
**Alternative diagnosis**	Any alternative diagnosis proposed by the reporting radiologist in the impression section	Recorded as free text

Supplementary Table S2: Examples of indeterminate CT reports in their original forms and after adjudicated, which reviewing radiologists agreed to categorization

Examples of original report impressions deemed "indeterminate for acute appendicitis"	Category after adjudication
No definite CT evidence of acute appendicitis. However, early acute appendicitis cannot be excluded. Please correlate with clinical context and consider repeat imaging if clinically suspicious.	Negative
Borderline enlarged appendix, about 6.5 mm, at proximal appendix with suspected appendicoliths. Air in the remaining part of appendix without definite wall thickening, hyperenhancement or periappendiceal fat stranding. Acute appendicitis is less likely. Please clinically correlate or follow up.	Negative
Dilated appendix secondary to colonic dilation.	Negative
Suspected acute appendicitis without complication. Please clinically correlate. (Findings: Enlarged fluid-filled appendix, measured about 1.3 cm, with wall hyperenhancement, wall thickening, minimal periappendiceal fluid and fat stranding.)	Positive
Probable acute appendicitis with suspected tiny appendicolith. No evidence of appendiceal rupture or abscess. (Findings: Appendiceal diameter = 7 mm. Mild wall hyperenhancement and periappendiceal fat stranding.)	Positive
Mild thickening and enhancing wall of appendix, diameter up to 9 mm at appendiceal base. Possible early acute appendicitis.	Positive

Supplementary Table S3: Final lexicon-based schemes for expressing certainty in the diagnosis of acute appendicitis adapted from Leite et al. for this investigation

Original category	Category used in this study	Appendiceal diameter (mm)*	Entirely gas-filled appendix	Wall thickening	Wall hyperenhancement	Fat stranding	Other findings
**Excludes**	**Negative**	<6	Any	Any	Any	Any	Any
**Excludes**	**Negative**	6-10	Yes	No	No	No	Any
**Possible**	**Possible**	6-10	No	No	No	No	No
**N/A**	**Possible**	6-10	No	No	No	No	Yes
**N/A**	**Possible**	6-10	Any	Yes	No	No	Any
**N/A**	**Possible**	6-10	Any	No	Yes	No	Any
**N/A**	**Possible**	6-10	Any	No	No	Yes	Any
**Probable**	**Probable**	6-10	Any	Yes	Yes	No	Any
**N/A**	**Probable**	6-10	Any	Yes	No	Yes	Any
**N/A**	**Probable**	6-10	Any	No	Yes	Yes	Any
**Definite**	**Positive**	6-10	Any	Yes	Yes	Yes	Any
**Definite**	**Positive**	>10	Any	Any	Any	Any	Any

*6 mm was revised to 7 mm in the 7-mm version.

Supplementary Table S4: Diagnostic test parameters of four methods of categorizing computed tomography reports for likelihood of acute appendicitis based on different grouping schemes*(n= 983)

	TP	FP	FN	TN	Sn	Sp	PLR	NLR	PPV	NPV	Acc
**Original report**											
**Negative vs. non-negative**	389	115	4	475	99.0 (97.4, 99.7)	80.5 (77.1, 83.6)	5.1 (4.3, 6.0)	0.01 (0.01, 0.03)	77.2 (74.2, 80.0)	99.2 (97.8, 99.7)	87.9 (85.7, 89.9)
**Positive vs. non-positive**	335	17	58	573	85.2 (81.3, 88.6)	97.1 (95.4, 98.3)	29.6 (18.5, 47.4)	0.2 (0.1, 0.2)	95.2 (92.5, 97.0)	90.8 (88.6, 92.6)	92.4 (90.5, 94.0)
**Adjudicated report**											
**Negative vs. non-negative**	388	83	5	507	98.7 (97.1, 99.5)	85.9 (82.9, 88.5)	7.02 (5.75, 8.57)	0.02 (0.01, 0.04)	82.4 (78.7, 85.6)	99.0 (97.7, 99.6)	91.0 (89.1, 92.7)
**Positive vs. non-positive**	335	17	58	573	85.2 (81.4, 88.4)	97.1 (95.4, 98.2)	29.58 (18.5, 47.4)	0.15 (0.12, 0.19)	95.2 (92.4, 97.0)	90.8 (88.3, 92.8)	92.4 (90.5, 93.9)
**Lexicon-based diagnostic scheme (6-mm version)**											
**Negative vs. non-negative**	393	389	0	201	100.0 (99.1, 100.0)	34.1 (30.3, 38.1)	1.5 (1.4, 1.6)	<0.01 (0.00, 0.03)	50.3 (48.8, 51.7)	100.0 (98.2, 100.0)	60.4 (57.3, 63.5)
**Low vs. high likelihood**	345	54	48	536	87.8 (84.1, 90.9)	90.9 (88.2, 93.1)	9.6 (7.4, 12.4)	0.1 (0.1, 0.2)	86.5 (83.2, 89.2)	91.8 (89.5, 93.6)	89.6 (87.6, 91.5)
**Positive vs. non-positive**	296	33	97	557	75.3 (70.8, 79.5)	94.4 (92.2, 96.1)	13.5 (9.6, 18.9)	0.3 (0.2, 0.3)	90.0 (86.5, 92.6)	85.2 (82.8, 87.2)	86.8 (84.5, 88.8)
**Lexicon-based diagnostic scheme (7-mm version)**											
**Negative vs. non-negative**	385	217	8	373	98.0 (96.0, 99.1)	63.2 (59.2, 67.1)	2.7 (2.4, 3.0)	0.03 (0.02, 0.06)	64.0 (61.5, 66.4)	97.9 (95.9, 98.9)	77.1 (74.4, 79.7)
**Low vs. high likelihood**	344	47	49	543	87.5 (83.9, 90.6)	92.0 (89.6, 94.1)	11.0 (8.3, 14.5)	0.1 (0.1, 0.2)	88.0 (84.7, 90.6)	91.7 (89.5, 93.5)	90.2 (88.2, 92.0)
**Positive vs. non-positive**	296	32	97	558	75.3 (70.8, 79.5)	94.6 (92.4, 96.3)	13.9 (9.9, 19.5)	0.3 (0.2, 0.3)	90.2 (86.8, 92.9)	85.2 (82.9, 87.3)	86.9 (84.6, 88.9)

*Original reports (3 probabilities of appendicitis) were regrouped to two categories of either A) negative vs. indeterminate plus positive ("negative vs. non-negative", or B) negative plus indeterminate vs. positive ("positive vs. non-positive"). The rest of reports (4 probabilities of appendicitis) were regrouped to two categories of A) negative vs. the rest ("negative vs. non-negative"), B) negative plus possible ("low likelihood") vs. probable plus positive ("high likelihood"), and C) positive vs. the rest ("positive vs. non-positive").

Likelihood ratios were reported to one decimal place. When values were <0.10, additional decimal precision was used, and values approaching zero were expressed as <0.01 to avoid misinterpretation due to rounding.

Supplementary Table S5. Expanded univariable and multivariable multinomial logistic regression analyses for factors associated with CT report category in adults suspected of acute appendicitis (reference category: indeterminate report)

Predictor	Outcome	Univariable OR (95% CI)	P value	Adjusted OR (95% CI)	P value
Age (per year)	Negative	1.01 (1.00–1.02)	0.262	1.03 (1.01–1.06)	0.008
	Definite	1.02 (1.01–1.03)	0.001	1.01 (0.99–1.02)	0.288
Appendix diameter (per mm)	Negative	0.46 (0.41–0.53)	<0.001	0.61 (0.49–0.76)	<0.001
	Definite	1.48 (1.35–1.63)	<0.001	1.47 (1.30–1.68)	<0.001
Male sex	Negative	1.32 (0.84–2.09)	0.233	1.17 (0.42–3.28)	0.760
	Definite	2.68 (1.68–4.27)	<0.001	1.89 (1.02–3.51)	0.045
**No alternative diagnosis**	Negative	0.25 (0.16–0.38)	<0.001	0.67 (0.28–1.61)	0.365
	Definite	7.60 (4.15–13.93)	<0.001	7.33 (3.11–17.29)	<0.001
**No wall thickening**	Negative	226.67 (54.00–951.46)	<0.001	18.19 (2.88–115.07)	0.002
	Definite	0.48 (0.31–0.73)	0.001	1.07 (0.59–1.96)	0.819
**No wall hyperenhancement**	Negative	259.32 (61.79–1088.37)	<0.001	147.74 (23.32–935.93)	<0.001
	Definite	0.44 (0.29–0.67)	<0.001	0.47 (0.26–0.88)	0.017
**No periappendiceal fat stranding**	Negative	840.31 (114.12–6187.79)	<0.001	277.51 (32.00–2406.86)	<0.001
	Definite	0.16 (0.09–0.27)	<0.001	0.34 (0.16–0.73)	0.005
**No appendicolith**	Negative	5.89 (2.93–11.85)	<0.001	5.57 (1.34–23.11)	0.018
	Definite	0.55 (0.32–0.96)	0.033	0.71 (0.32–1.58)	0.405
**Department group**					
ER/duty doctor vs IPD/ICU	Negative	0.79 (0.44–1.43)	0.442	1.05 (0.33–3.37)	0.937
	Definite	2.45 (1.23–4.88)	0.011	2.20 (0.81–5.95)	0.121
OPD vs IPD/ICU	Negative	1.08 (0.57–2.03)	0.815	1.45 (0.43–4.94)	0.554
	Definite	2.42 (1.17–5.03)	0.017	3.75 (1.32–10.66)	0.013
**Prior ultrasonography**	Negative	1.22 (0.51–2.89)	0.655	-	-
	Definite	1.63 (0.64–4.19)	0.310	-	-

Abbreviations: OR, odds ratio; aOR, adjusted odds ratio; CI, confidence interval. Adjusted multinomial logistic regression included age, sex, appendix diameter, alternative diagnosis on CT, wall thickening, wall hyperenhancement, periappendiceal fat stranding, appendicolith, department group, ultrasound prior to CT, white blood cell count >10,000 cells/µL, neutrophil percentage >75%, lymphocyte percentage, and neutrophil-to-lymphocyte ratio >4.7 as candidate variables. Prior ultrasonography was not retained in the final multivariable model because it was not significantly associated with CT report category. Inclusion of prior ultrasonography and laboratory variables did not materially alter the main associations identified in the original model.
